# Molecular and morphological identification of *Biomphalaria* species from the state of São Paulo, Brazil

**DOI:** 10.3897/zookeys.668.10562

**Published:** 2017-04-12

**Authors:** Raquel Gardini Sanches Palasio, Marisa Cristina de Almeida Guimarães, Fernanda Pires Ohlweiler, Roseli Tuan

**Affiliations:** 1 Department of Epidemiology, Faculty of Public Health, University of São Paulo, Pinheiros, SP, Brazil; 2 Biochemistry and Molecular Biology Laboratory, Superintendency for the Control of Endemic Diseases, Rua Paula Sousa, 166, Luz, São Paulo, SP, Brazil; 3 Malacology Laboratory, Regional Service SR-02, Superintendency for the Control of Endemic Diseases, São Vicente, Rua João Ramalho, 587, Centro, SP, Brazil; 4 Malacology Laboratory, Special Programs Division SR-01, Superintendency for the Control of Endemic Diseases, Rua Cardeal Arcoverde, 2878 - Pinheiros, SP, Brazil

**Keywords:** *Biomphalaria*, COI, DNA barcoding, morphological taxonomy, schistosomiasis, species identification

## Abstract

DNA barcoding and morphological characters were used to identify adult snails belonging to the genus *Biomphalaria* from 17 municipalities in the state of São Paulo, Brazil. The DNA barcode analysis also included twenty-nine sequences retrieved from GenBank. The final data set of 104 sequences of the mitochondrial cytochrome oxidase I (COI) gene was analyzed for K2P intraspecific and interspecific divergences, through tree-reconstruction methods (Neighbor-Joining, Maximum Likelihood and Bayesian inference), and by applying different models (ABGD, bPTP, GMYC) to partition the sequences according to the pattern of genetic variation. Twenty-seven morphological parameters of internal organs were used to identify specimens. The molecular taxonomy of *Biomphalaria* agreed with the morphological identification of specimens from the same collection locality. DNA barcoding may therefore be a useful supporting tool for identifying *Biomphalaria* snails in areas at risk for schistosomiasis.

## Introduction

Brazil contains one of the richest faunas of freshwater snails of the genus *Biomphalaria* (Agostinho et al. 2005, Scholte et al. 2012). The state of São Paulo, in southeast Brazil, is of enormous epidemiological importance, as all the three Neotropical intermediate hosts of *Schistosoma mansoni* (Sambon, 1907), *Biomphalaria glabrata* (Say, 1818), *B. straminea* (Dunker, 1848), *B. tenagophila* (d’Orbigny, 1835), are distributed in streams, ponds, dams and reservoirs in this municipality. *Biomphalaria occidentalis* (Paraense, 1981), *B. peregrina* (d’Orbigny, 1835), *B. intermedia* (Paraense & Deslandes, 1962), *B. oligoza* (Paraense, 1975) and *B. schrammi* (Crosse, 1864) are also distributed in São Paulo state (Vaz 1989; Teles 2005; Ohlweiler et al. 2010).

Identification of *Biomphalaria* specimens to the species level and analysis of infection by *S. mansoni* are key elements of surveillance strategies for schistosomiasis control and elimination (PAHO 1968, WHO 2013). Shell morphology is of limited use for identifying different species of snails in this genus (Paraense 1966; Jarne and Théron 2001), and therefore the anatomical characters described by Paraense (1961, 1975, 2001) are used instead. However, identification of *Biomphalaria* solely based on morphological characters is constrained by phenotypic plasticity, the limited descriptions of cryptic species, and the difficulty in applying species-diagnostic characters to juvenile specimens (Carvalho et al. 2008; Teodoro et al. 2010). The issue of how useful molecular tools may be in the identification of *Biomphalaria* snails has become particularly important in recent years as there is consensus among malacologists that morphological identification using internal anatomical parameters is susceptible to error, especially when the snails being analyzed belong to complexes of morphologically similar species (Paraense 1972, 1974, 1988; Spatz et al. 1999; Vidigal et al. 2000). To overcome these limitations and difficulties associated with traditional taxonomy, various methodologies based on molecular markers have been developed.

PCR-RFLP analysis of mitochondrial and nuclear genes (Spatz et al. 1999; Vidigal et al. 1998, 2000; Caldeira et al. 2000, 2009), fingerprinting techniques using nonspecific primers (Abdel-Hamid et al. 1999; Al-Quraishy et al. 2014) and sequence analysis of COI and r16RNA genes and ITS-1 and ITS-2 sequences (Woolhouse and Chandiwana 1989; Langand et al. 1998; Vidigal et al. 2000; Campbell et al. 2000; DeJong et al. 2003; Wethington et al. 2007; Tuan and Santos 2007; Tuan et al. 2012) have all produced results that allowed significant genetic differences in species and populations to be identified.

When used in conjunction with bioinformatics tools and sequence databases, DNA barcoding routinely facilitates the identification of biological species (Ratnasingham and Hebert 2007; Casiraghi et al. 2010). This technique is based on the polymorphism of a short region (approximately 600 bp long) of the mitochondrial cytochrome c oxidase 1 (COI) gene (Hebert et al. 2003). DNA barcode includes a series of strategies for delimiting species into molecular operational taxonomic units (MOTUs) using a combination of laboratory and bioinformatics methods (Fontaneto et al. 2013). The most important strategies for identifying MOTUs include analysis of intraspecific and interspecific genetic distances, and analyses based on population and phylogenetic models. These approaches include (ABGD) (Puillandre et al. 2012) and the barcode index number (BIN) system (Ratnasingham and Hebert 2013), which use algorithms based on the partition of molecular data according distance methods, and the generalized mixed Yule coalescent (GMYC) method (Fujisawa and Barraclough 2013) and Bayesian Poisson Tree Processes (bPTP) method (Zhang et al. 2013).

DNA barcoding has been used to augment morphological identification of *Bulinus* in Africa (Kane at al. 2008; Stothard et al. 2013; Standley et al. 2014), and yielded better results than identifications based on shell characters. Although there are over 500 COI sequences in GenBank from snails of the genus *Biomphalaria* found in African and Neotropical regions, most DNA barcoding studies use African species. There is therefore a dearth of knowledge about the effectiveness of DNA barcoding in taxonomic identification of Neotropical species of *Biomphalaria* (Standley et al. 2011; Tuan et al. 2012).

Here, we investigate the utility of analysis of distributions of intraspecific and interspecific COI divergences based on genetic distances, tree reconstruction methods based on Bayesian inference, Maximum Likelihood (ML), and K2P-Neighbor-Joining (NJ) grouping of sequences, and the ABGD, GMYC and bPTP methods for delimitation of *Biomphalaria* species in conjunction with schistosomiasis field surveys.

## Materials and methods

### Experimental design

**Figure 1.**
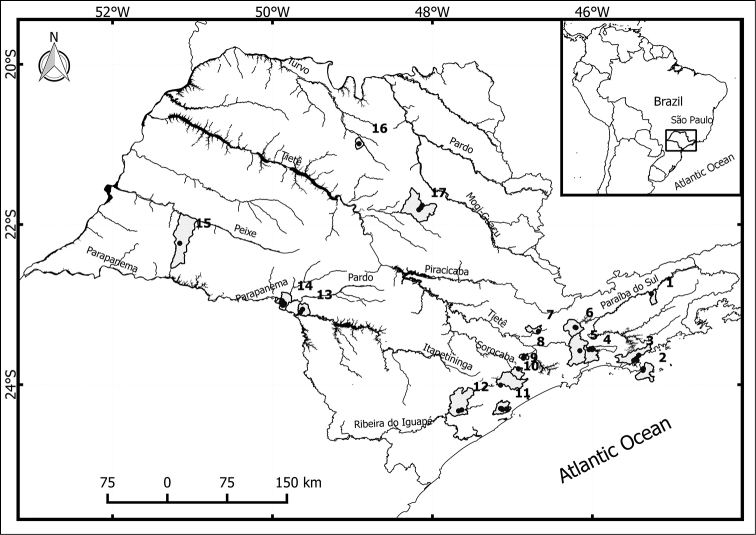
Locations of the 17 municipalities in São Paulo (Brazil) where the snails were collected. **1** Aparecida **2** Ilhabela **3** Caraguatatuba **4** Biritiba Mirim **5** Mogi das Cruzes **6** Santa Isabel **7** Franco da Rocha **8** Embu das Artes **9** São Lourenço da Serra **10** Juquitiba **11** Itariri **12** Juquiá **13** Ipaussu **14** Ourinhos **15** Martinópolis **16** Novais **17** Araraquara (coordinates are detailed in Table 1).

Planorbids were collected in 17 municipalities in the state of São Paulo, Brazil between May 2012 and January 2013 (Fig. 1). The collection points were georeferenced with a Garmin ETrex Summit® GPS (Table 1).

**Table 1.** Collection localities, sample information, and GenBank accession numbers for COI sequences used in this study.

Sample Sites/ Country	Map locality	Municipality	Latitude; Longitude	COI sequence	GenBank accession number
São Paulo, Brazil	1	Aparecida	22°51'52.0"S; 45°15'46.0"W	589, 588, 591	KF926184, KF926196, KF926186
	2	Ilhabela	23°49'17.5"S; 45°22'01.4"W	564,555	KF926191, KF926187
			23°47'56.4"S; 45°21'44.0"W	593,554	KF926213, KF926212
	3	Caraguatatuba	23°37'55.7"S; 45°25'08.7"W	563	KF926218
			23°37'59.6"S; 45°25'11.4"W	517	KF926105
			23°38'04.2"S; 45°25'14.7"W	579, 580	KF926221, KF926217
			23°38'3.25"S; 45°25'14.3"W	516	KF926106
			23°40'26.1"S; 45°26'54.3"W	592	KF926215
			23°40'42.2"S; 45°27'18.5"W	568	KF926214
			23°41'34.8"S; 45°26'58.1"W	569	KF926216
			23°41'46.4"S; 45°28'57.9"W	565, 571	KF926219, KF926220
			23°41'49.5"S; 45°26'30.8"W	523	KF926222
	4	São Paulo	23°33'43.0"S; 45°59'66.0"W	551	KF926204
			23°33'44.0"S; 46°02'35.0"W	548, 549	KF926203, KF926205
	5		23°33'95.0"S; 46°09'24.0"W	547	KF926202
	6	Santa Isabel	23°17'16.8"S; 46°12'16.1"W	544	KF926174
			23°17'00.2"S; 46°12'59.1"W	545, 546, 550, 552	KF926177, KF926189, KF926195, KF926190
	7	Franco da Rocha	23°20'02.0"S; 46°40'28.0"W	-	
	8	Embu das Artes	23°38'50.5"S; 46°51'11.3"W	524	KF926197
			23°40'08.5"S; 46°51'41.7"W	640	KF926198
	9	Embu-Guaçu	23°48'11.0"S; 46°55'27.0"W	630	KF926201
	10	Juquitiba	24°00'21.0"S; 47°08'52.0"W	-	
	11	Itariri	24°17'53.6"S; 47°08'55.0"W	537	KF926188
			24°17'55.0"S; 47°08'06.8"W	536	KF926211
			24°18'26.3"S; 47°03'58.9"W	618	KF926207
			24°18'39.9"S; 47°07'31.4"W	503	KF926206
			24°18'11.8"S; 47°04'04.1"W	532, 627, 534	KF926209, KF926185, KF926208
			24°18'13.5"S; 47°04'31.7"W	535	KF926210
São Paulo, Brazil	12	Juquiá	24°18'55.1"S; 47°37'58.6"W	650, 651, 653	KT225577, KT225578, KT225579
			24°19'39.5"S; 47°40'25.0"W	655	KT225580
	13	Ipaussu	23°05'39.6"S; 49°39'01.5"W	756, 761, 755	KX354441-KX354442, KX354440
	14	Ourinhos	22°57'00.2"S; 49°52'33.1"W	764	KX354435
			22°58'02.5"S; 49°52'27.1"W	572, 543, 573	KF926181, KF926182, KF926183
			22°58'03.4"S; 49°52'28.9"W	735, 733, 766	KX354437-KX354438, KX354433
			22°59'08.0"S; 49°50'59.9"W	577	KF926192
			22°58'29.5"S; 49°53'29.4"W	538, 578	KF926165, KF926193
			23°00'24.8"S; 49°51'48.7"W	739	KX354444
			23°00'32.2"S; 49°52'21.9"W	763, 765	KX354436, KX354434
			23°00'11.5"S; 49°51'41.4"W	747	KX354443
			22°57'11.6"S; 49°52'41.9"W	636, 540	KF926194, KF926166
			22°57'11.6"S; 49°52'41.9"W	575, 542	KF926168, KF926167
			22°59'42.4"S; 49°52'27.6"W	541	KF926178
	15	Martinópolis	22°14'04.4"S; 51°09'36.4"W	581, 582	KX354445, KF926180
	16	Novais	20°59'30.0"S; 48°55'05.0"W	570, 586, 587	KF926179, KF926169, KF926171
	17	Araraquara	21°45'37.9"S; 48°07'40.1"W	595, 599, 601	KF926170, KF926173, KF926172
			21°47'30.3"S; 48°08'41.1"W	594, 596	KF926199, KF926200
			21°48'57.1"S; 48°10'13.1"W	602	KF926175
Argentina		*ARG_1, ARG_2, ARG_3, ARG_4*			JN621901, JN621902 JN621903, GU168593
Brazil		*BRA_1*			AF199090
		RS_*BRA_2*, RS_*BRA_3*, RS_*BRA_4*, MG_*BRA_5*, *BRA*_6, *BRA_7*, *BRA_8*, *BRA*_9			KF926107, KF926108 KF926109 AF199094 AF199091 , AF199092, AF199095 , AF199096
		RS_*BRA_10*			KX354439
		*BRA_*11			AF199084
Brazil		RS_*BRA_12*, RS_*BRA_13*, RS_*BRA_14*, RS_*BRA_15*, RS_*BRA_16*			KF926155-KF926156 KX354446-KX354447 KX354448
		*BRA*_*17* RS_BRA_18, RS_BRA_19			AF199089 EF433576, NC010220
Egypt		*EGY_2, EGY_1*			DQ084823 AF199111
Hong Kong		*HKG*			AF199085
M-Line *					AY380567
Puerto Rico		*PUR*			DQ084824
Venezuela		*VEN*			AF199093

^*^M-Line refers to a laboratory strain of *Biomphalaria glabrata* derived from a Puerto Rico pigmented snail and an albino Brazilian snail (Mulvey and Bandoni 1994).

Samples were collected from freshwater habitats in the Paranapanema, Tietê, Ribeira do Iguape and Paraíba do Sul River basins and the northern coast of São Paulo that had been previously surveyed and classified according to the risk for schistosomiasis transmission as part of a program to monitor snails that are intermediate hosts of *S. mansoni* (*Biomphalaria*).

In accordance with the methods described in the Brazilian Ministry of Health Schistosomiasis Surveillance and Control Program (Ministry of Health 2008), snails were collected at sampling stations in each freshwater body and grouped into batches according to their origin. Most of the snails in each batch were then exposed to artificial light in the laboratory to determine whether they were infected with cercariae. At least two specimens from each batch were used for morphological analysis and at least two for the DNA barcode analysis.

DNA barcoding was applied to 75 adult snails taken from samples collected in the field. Only snails that did not have any parasite larvae in their digestive gland and ovotestis were used for molecular identification. Shells were removed by compressing each snail between two slides. After removing the shell fragments, each crushed snail was transferred to a clean Petri dish. The portion of the cephalopodal mass corresponding to the foot was excised under a stereo microscope with forceps and scissors and used as starting material for isolation of total DNA. To maximize the efficiency of genomic DNA purification we used fresh material that had not been fixed. Each specimen was then dissected and identified to the species level based on the presence or absence of the renal ridge and the most informative characters of the male and female copulatory organs. DNA barcoding was carried out in a blind fashion, i.e., without prior knowledge of the general morphological characteristics identified in the animal.

An additional 118 adult specimens were taken from the same field samples (at least two per batch) and scored for 27 morphological characters used by Paraense (1975, 1981, 1984, 2001) in his descriptions of Neotropical species of the genus *Biomphalaria*. The soft parts were removed from the shell after placing the snails in 70°C for 40 seconds and then fixing them in Railliet-Henry’s solution (distilled water 930 mL, sodium chloride 6 g, formalin 50 mL and glacial acetic acid 20 mL). After at least 24 hours of fixation, the specimens were dissected following Deslandes’ (1951) protocols to examined the renal tube and reproductive system. Specimens were not anesthetized prior to fixation to ensure that the procedure followed was the same as that used in our malacology laboratories.

The longitudinal renal ridge is considered the gold-standard character for differentiating *B. glabrata* (Paraense and Deslandes 1959) from other species in the genus, in which the ridge is absent. The anterior and posterior regions of the vagina were examined. The proportions for the diameter and length of the oviduct were based on the nidamental gland; for the diameter of the uterus, the cephalic portion of the nidamental gland was used, for the length of the uterus, the posterior region of the vagina; for the length of the spermathecal duct, the body of the spermatheca; and for the length of the anterior region of the vagina, the posterior region of the vagina. The relative proportions of the organs or structures were used for comparisons together with the shell and mantle pigmentation pattern.

### DNA extraction, amplification and sequencing

DNA isolation was carried out with the DNeasy Tissue Kit (Qiagen®). A fragment of the COI gene (~600 bp) was amplified with the LCO/HCO primers (Folmer et al. 1994). Polymerase chain reaction (PCR) was carried out in a total volume of 50 µL and the following reaction mixture: 10-100 ng of DNA, 0.2 mM of each dNTP, 0.10 μM of each primer and 1 U of Taq DNA polymerase in the supplied reaction buffer. The cycling conditions consisted of an initial 3 min step at 95°C for denaturation; 25 cycles of 1 min at 95°C, 1 min at 47°C and 1 min 30 s at 72°C and a final extension step of 7 min at 72°C (Tuan et al. 2012). PCR products were purified with a Qiagen purification kit and then sequenced in the Biotechnology Center at the Butantan Institute in an ABI3100 automated sequencer (Applied Biosystems®).

### Molecular data analysis

The electropherograms obtained from forward and reverse sequencing of each specimen were corrected using CHROMAS (Technelysium Pty Ltd.) and then aligned with CLUSTALX version 1.8 (Thompson et al. 1997). The aligned sequences were edited with BIOEDIT version 7.0 (Hall 1999), and the general polymorphism of the sequences was calculated in DNAsp version 5 (Librado and Rozas 2009).

The final alignment consisted of a matrix of 75 COI sequences from the collected specimens (36 *B. tenagophila*, 12 *B. occidentalis*, 10 *B. glabrata*, 9 *B. straminea*, 1 *B. intermedia*, 7 *B. peregrina)* and 29 COI sequences of *Biomphalaria* from other Neotropical areas that were retrieved from GenBank (Table 1).

Intraspecific and interspecific genetic distances (Kimura 1980) were calculated by pairwise comparison of the sequences of all the individuals using the Kimura 2-parameter (K2P) method with the MEGA 6 (Molecular Evolutionary Genetics Analysis) package (Tamura et al. 2013). Three tree-based methods were performed for phylogenetic reconstructions. The K2P distance matrix was used to reconstruct a Neighbor-Joining (NJ) tree. MEGA 6 was also used to perform Maximum Likelihood analysis. In the ML analysis, the GTR+I+G model of sequence evolution was chosen using the Akaike information criterion as implemented in MODELTEST 2.3 (Nylander 2004). The reliability of NJ and ML topologies was evaluated using bootstrap support with 1000 replicates. The parameters estimated by MODELTEST were also used in a Bayesian Markov-Chain Monte Carlo (MCMC) analysis in MRBAYES 3.1 (Huelsenbeck and Ronquist 2001; Ronquist and Huelsenbeck 2003). Two simultaneous independent searches were run for 1.5 x 106 generations, with trees saved every 100 generations, and the first 1.500 sampled trees of each search discarded as “burn-in”.

The barcode gap analysis was performed with the ABGD (Puillandre et al. 2012), bPTP (Zhang et al. 2013) and GMYC methods (Fujisawa and Barraclough 2013). ABGD, bPTP and GMYC were run on the http://www.abi.snv.jussieu.fr/ public/abgd/, http://species.h-its.org/ and http://species.h-its.org/gmyc/ web servers, respectively, using default parameters.

All the molecular analysis was performed on the 104 sequences (39 *B. tenagophila*, 23 *B. glabrata*, 13 *B. occidentalis*, 11 *B. straminea*, 12 *B. peregrina*, 1 *B. intermedia*, and 5 sequences from *B. tenagophila guaibensis*) (Table 1). *Biomphalaria oligoza* was excluded from the analysis because we were unable to amplify its DNA.

## Results

### Morphological analysis

The morphological identifications of the 118 adult snails that were studied are presented in Table 2. The results of morphological analysis revealed the following: **Shell**: the presence of a carina, the shape of the whorls and the shape of the shell aperture distinguished *B. tenagophila* and *B. occidentalis* from the other species in the group. **Renal tube**: The presence of renal ridge was observed in all the *B. glabrata* specimens studied. **Pigmentation of the mantle**: adult specimens of *B. tenagophila*, *B. glabrata* and *B. occidentalis* had more uniform pigmentation than the four other species studied, which had blotchy pigmentation. **Reproductive system**: the presence of a vaginal pouch in *B. tenagophila* and its absence in *B. occidentalis* differentiates these two species. *Biomphalaria straminea* and *B. intermedia* had marked variation in the posterior region of the vagina; in the former, the corrugation in this region was markedly wavy while in the latter it was swollen.

**Table 2.** Morphological characters used to identify 118 *Biomphalaria* specimens from the state of São Paulo.

Morphological character	*B. glabrata* (n= 9)	*B. tenagophila* (n= 56)	*B. occidentalis* (n= 18)	*B. oligoza* (n= 10)	*B. peregrina* (n= 9)	*B. intermedia* (n=10)	*B. straminea* (n= 6)
Carinate shell	Absent	Present	Present	Absent	Absent	Absent	Absent
Shape of the whorls on the shell	Rounded	Angular	Angular	Rounded	Rounded	Rounded	Rounded
Shell aperture	Rounded	Transverse, low or deltoid	Transverse, low or deltoid	Rounded	Rounded slightly to the right	Rounded	Rounded
Mantle pigmentation	Tends to be homogeneous	Tends to be homogeneous	Tends to be homogeneous	Spotted or blotchy	Spotted or blotchy	Spotted or blotchy	Spotted or blotchy
Longitudinal renal ridge	Present	Absent	Absent	Absent	Absent	Absent	Absent
Number of ovotestis diverticula	More than 100	More than 100	More than 100	18 to 37	More than 100	Around 60	More than 100
Shape of the ovotestis diverticula	Elongate, simple or subdivided	Elongate, simple or subdivided	Elongate, simple or subdivided	Bulging and simple	Elongate, simple or subdivided	Elongate, simple or subdivided	Elongate, simple or subdivided
Differentiation of the ovotestis diverticula	Weakly differentiated	Weakly differentiated	Weakly differentiated	Well differentiated	Well differentiated	Well differentiated	Well differentiated
Diameter of the oviduct	Narrow	Narrow	Narrow	Wide	Narrow	Wide	Wide
Length of the oviduct	Long	Long	Long	Short	Long	Long	Short
Appearance of the oviduct pouch	Clearly defined	Clearly defined	Clearly defined	Bulky	Clearly defined	Clearly defined	Bulky
Diameter of the uterus	Narrow	Narrow	Narrow	Wide	Wide	Narrow	Wide
Length of the uterus	Long	Long	Long	Short	Short	Long	Long
Length of the anterior region of the vagina	Long	Long	Long	Short	Short	Long	Long
Corrugation on the dorsal wall of the posterior region of the vagina	Absent	Absent	Absent	Absent	Absent	Present	Present
Type of vaginal Corrugation	-	-	-	-	-	Swollen	Strongly wavy
Vaginal pouch on the ventral wall of the posterior region of the vagina	Present	Present	Absent	Present	Present	Present	Absent
Shape of the vaginal pouch	Elongate	Bulging	_	Elongate	Elongate	Elongate	_
Appearance of the vaginal pouch	Clearly defined	Clearly defined	_	Discrete	Clearly defined	Discrete	_
Length of the spermathecal duct	Long	Long	Long	Short	Short	Long	Long
Shape of the prostate diverticula	Tree-like	Tree-like	Tree-like	Simple or subdivided	Tree-like	Tree-like	Tree-like
Number of prostate diverticula	Around 30	Around 30	Around 20	1 to 4	Around 13	Around 13	Around 20
Length of the penial sheath	Approx. the same length as the prepuce	Approx. the same length as the prepuce	Shorter than the prepuce	Approx. the same length as the prepuce	Longer than the prepuce	Approx. the same length as the prepuce	Longer than the prepuce
Diameter of the penial sheath	Narrow	Narrow	Narrow	Wide	Wide	Wide	Wide
Shape of the prepuce	Free end is wider	Free end is wider	Same diameter along its whole length	Same diameter along its whole length	Free end is wider	Free end is wider	Free end is wider
Seminal vesicle extensions	Finger-like	Finger-like	Finger-like	Nodular	Finger-like	Finger-like	Finger-like
Appearance of the seminal vesicle	Developed	Developed	Poorly developed	Poorly developed	Developed	Developed	Developed

*Biomphalaria peregrina* differed from the species in the *B. straminea* complex (*B. straminea* and *B. intermedia*) in the width of the oviduct, the length of the uterus, the length of the spermathecal duct and the length of the anterior region of the vagina. *Biomphalaria intermedia* differed from *B. straminea* in the number of ovotestis diverticula, the length of the oviduct, the presence of an oviduct pouch, the number of prostate diverticula and the width of the uterus.

*Biomphalaria oligoza*, *B. peregrina*, *B. intermedia* and *B. straminea* are differentiated by the number and shape of the ovotestis diverticula, appearence and size of seminal vesicle, the number and shape of the prostate diverticula, and the shape of the prepuce. The 27 morphological characters used to identify *Biomphalaria* are detailed in Table 2.

All these findings are in agreement with Paraense and Deslandes (1959), Paraense (1961, 1974, 1975, 1981, 1984).

### Molecular analysis

The final alignment matrix for the 104 sequences consisted of 549 characters including 25% polymorphic, 21% parsimony-informative and 12 unique sites (Table 3).

**Table 3.** Sample size (N), number of haplotypes (H), haplotype diversity (Hd), nucleotide diversity (π, Nei 1987, equation 10.5) and average number of nucleotide differences (K, Tajima 1983, equation A3) calculated in DNAsp v.5 (Librado and Rozas 2009) for a 549 bp region of the COI gene in the six *Biomphalaria* species and one *Biomphalaria* subspecies.

Species	N	H	Hd	π	K
*Biomphalaria*	104	36	0.946	0.06805	
*B. straminea*	11	6	0.836	0.01199	6.582
*B. occidentalis*	13	1	0.000	0.00000	0.000
*B. peregrina*	12	6	0.848	0.01954	10.727
*B. glabrata*	23	10	0.862	0.01914	10.506
*B. tenagophila*	39	11	0.803	0.01222	6.707
*B. t. guaibensis*	5	1	0.000	0.00000	0.000
*B. intermedia*	1	1	-	-	-

The K2P sequence divergence for intraspecific comparisons ranged from 0.0% to 4.0%, while for interspecific comparisons the corresponding figure varied from 4.0% to 12% (Table 4). The greatest intraspecific genetic distances were observed between specimens of *B. peregrina* from SP and Rio Grande do Sul (southern Brazil) (4.0%) and specimens of *B. glabrata* from Rio Grande do Sul and Puerto Rico (3.9%).

**Table 4.** Intraspecific and interspecific genetic distances (COI) generated using the Kimura 2-parameter model (K2P, Kimura 1980) in MEGA6 (Tamura et al. 2013).

Species 1	Species 2	Minimum distance	Mean distance	Maximum distance
Intraspecific				
*B. glabrata*		0.00	0.03	0.04
*B. tenagophila*		0.00	0.02	0.03
*B. straminea*		0.00	0.01	0.03
*B. occidentalis*		0.00	0.00	0.00
*B. peregrina*		0.00	0.02	0.04
*B. intermedia*		0.00	0.00	0.00
*B. t. guaibensis*		0.00	0.00	0.00
Interspecific				
*B. glabrata*	*B. tenagophila*	0.07	0.09	0.10
	*B. straminea*	0.07	0.09	0.10
	*B. occidentalis*	0.09	0.09	0.09
	*B. peregrina*	0.10	0.12	0.15
	*B. intermedia*	0.06	0.08	0.09
	*B. t. guaibensis*	0.07	0.09	0.09
*B. tenagophila*	*B. straminea*	0.08	0.09	0.10
	*B. occidentalis*	0.04	0.05	0.06
	*B. peregrina*	0.10	0.12	0.15
	*B. intermedia*	0.05	0.08	0.09
	*B. t. guaibensis*	0.04	0.04	0.05
*B. straminea*	*B. occidentalis*	0.09	0.09	0.10
	*B. peregrina*	0.09	0.01	0.10
	*B. intermedia*	0.05	0.05	0.06
	*B. t. guaibensis*	0.08	0.08	0.09
*B. occidentalis*	*B. peregrina*	0.10	0.11	0.13
	*B. intermedia*	0.08	0.08	0.08
	*B. t. guaibensis*	0.03	0.03	0.03
*B. peregrina*	*B. intermedia*	0.09	0.09	0.10
	*B. t. guaibensis*	0.10	0.12	0.13
*B. intermedia*	*B. t. guaibensis*	0.08	0.08	0.08

**Figure 2.**
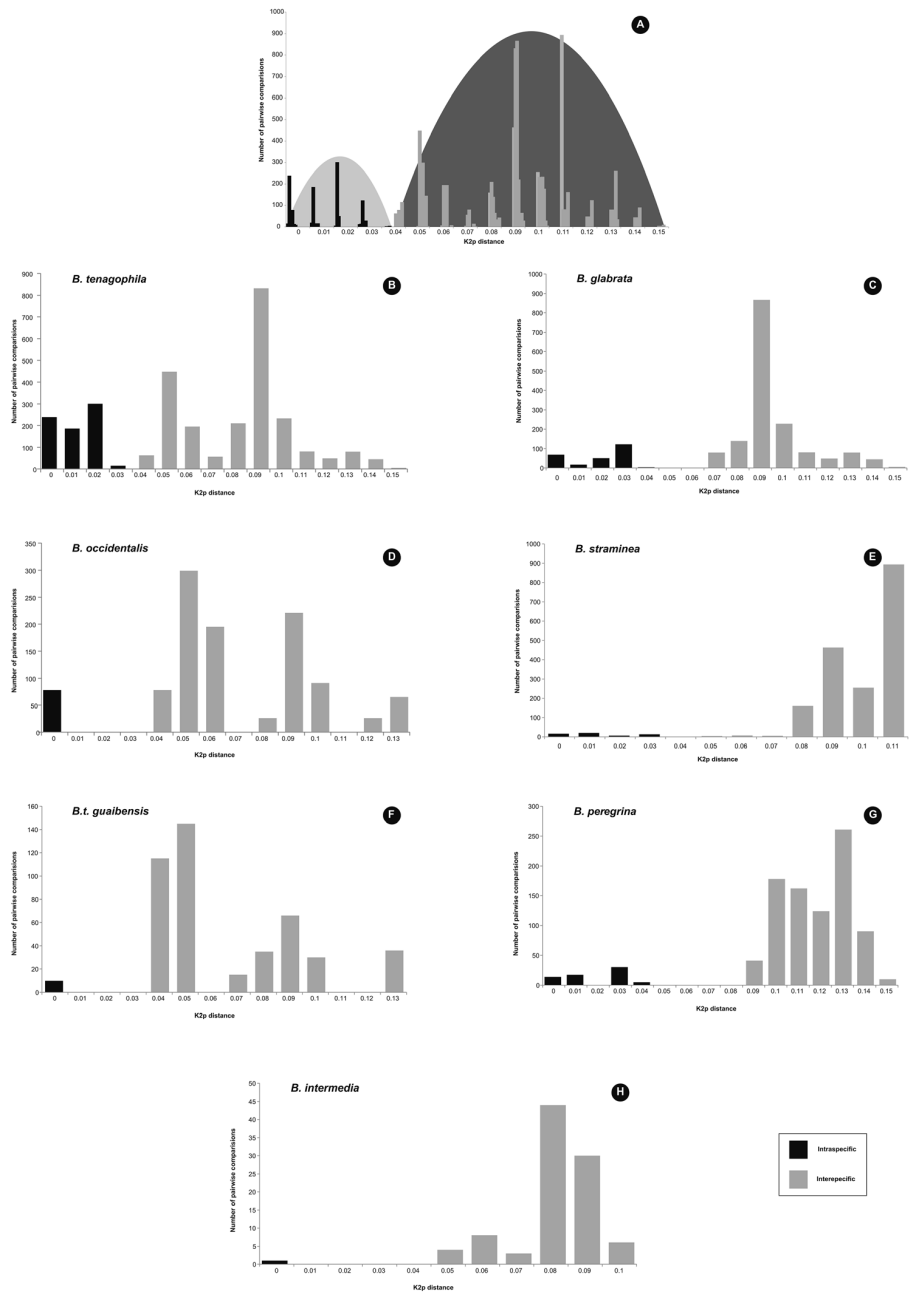
**A** histogram showing pairwise Kimura 2-parameter intraspecific and interspecific distances for 104 *Biomphalaria* cytochrome oxidase I sequences **B–H** pairwise distances between each species and the other taxa analyzed.

The frequency distribution of the 104 analyzed sequences indicates that although there were some extreme pairwise distances (>3%) in *B. glabrata*, *B. tenagophila*, *B. peregrina* and *B. straminea*; intraspecific and interspecific divergences did not overlap (Fig. 2A). Nevertheless, a typical barcode gap was not observed in this dataset. A closer inspection of the distances for each taxonomic group shows that there is a clear barcode gap between *B. glabrata*, *B. straminea*, *B. peregrina* and *B. intermedia*. There was no clear barcode gap between closely related *B. tenagophila*, *B. t. guaibensis* and *B. occidentalis* (interspecific distance 3-4%) (Fig. 2 C, D, E, F).

**Figure 3.**
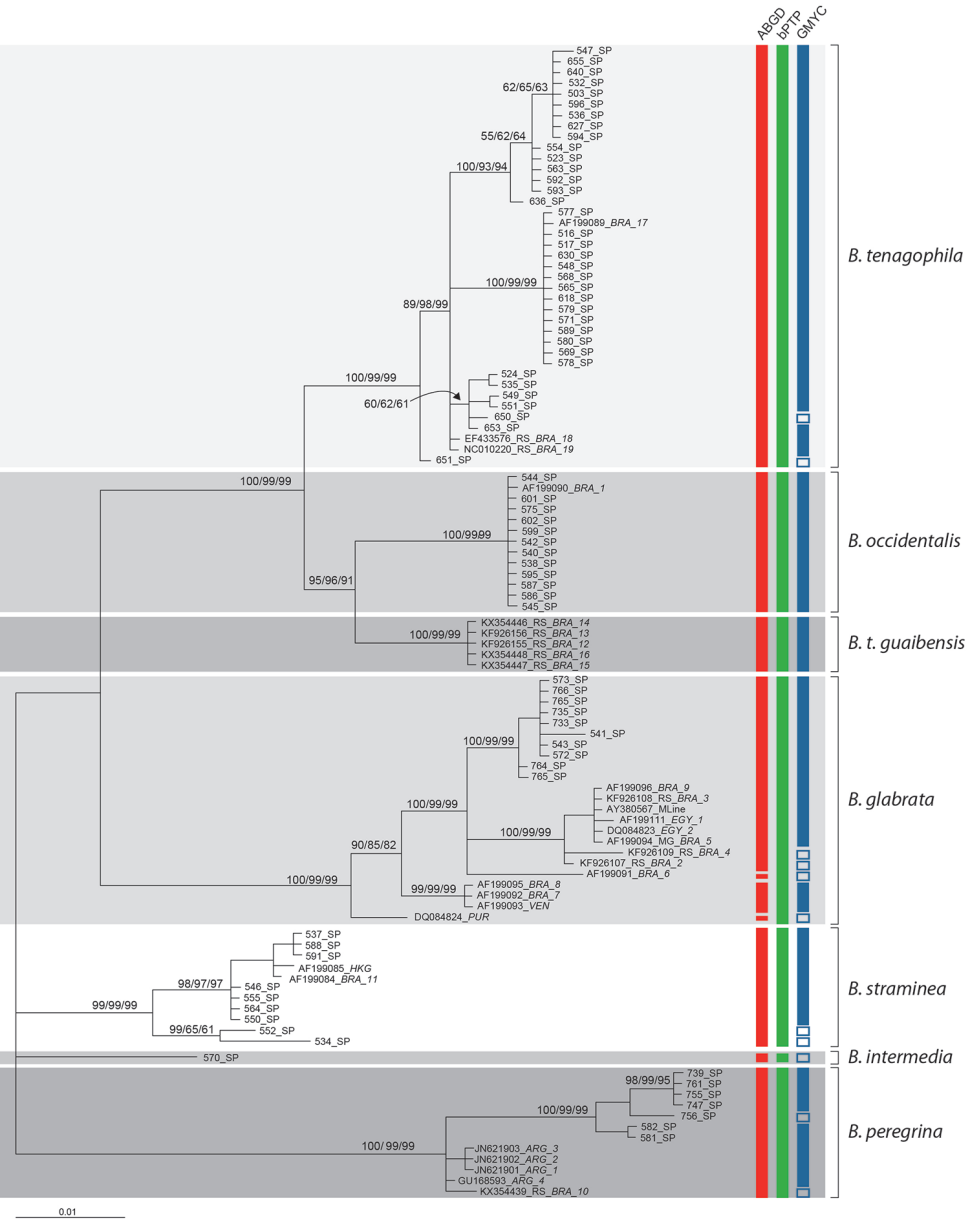
Bayesian phylogram. Support values for individual branches are given as Bayesian credibility/ML bootstrap/NJ bootstrap and are depicted above each node. The different shades of gray identify morphological species. The red, green and blue bars indicate species delimitations based on the distance-based (ABGD) and tree-based (bPTP and GMYC) models, respectively.

The total number of MOTUs within the same taxon (Fig. 3) varied depending on the model used to partition the COI data (GMYC, bPTP or ABGD). Only bPTP recovered all seven groups identified by traditional morphology. GMYC revealed various sequences that were not consistent with morphological identifications: *B. peregrina* sequences from Rio Grande do Sul (BRA_10/KX354439) and São Paulo (756/KX354441), *B. straminea* sequences from Santa Isabel (SP) and Itariri (SP) (552/KF926190, 534/KF926185), one *B. intermedia* sequence (570/KF926179), two *B. tenagophila* sequences from Juquiá (SP) and four *B. glabrata* sequences from GenBank (RS_BRA_2/KF926107, RS_BRA_4/KF926109, BRA_6/AF199091 and PUR/DQ084824).

When run using the default settings, ABGD recovered five different subunits of *B. glabrata*. This result may be explained by the pronounced genetic variation in this species, but the possibility that these subgroups represent cryptic taxa cannot be ruled out.

The trees generated by the Bayesian, ML and NJ methods (Fig. 3) delineated six well supported groups (posterior probabilities and bootstrap values ≥90) congruent with the current classification of *Biomphalaria*. The only *B. intermedia* sequence appeared in a distinct branch supported by low Bayesian and bootstrap values.

## Discussion

This study sought to determine the utility of DNA barcoding in delimiting species in freshwater snails of the genus *Biomphalaria*. The Bayesian, ML and NJ analyses (Fig. 3, Suppl. material 1) yielded trees with well-supported internal branches (≥90), resolving six out of the seven taxa as monophyletic groups.

The assessment of the potential of DNA barcode for species differentiation in *Biomphalaria* essentially revolves around the comparison of results of the morphological and molecular analysis of closely similar or taxonomically ambiguous species. In the case of the three taxa in the *B. tenagophila* complex, one character that is normally effective for specific identification is the vaginal pouch, which is present in *B. tenagophila* and *B. t. guaibensis* but not in *B. occidentalis*. (The anatomical features of these three taxa were illustrated by Tuan et al. 2012). Although we did not observe this in our material, in some specimens of *B. occidentalis* there is a slight projection of the ventral wall of the vagina (Paraense 1981), which raises questions regarding the distinctness of this taxon.

The intraspecific genetic distance within *B. tenagophila* showed values with a range from 0 to 3% (Table 4, Figs 2, 3). A high level of genetic divergence within this species was obtained for sequences associated with specimens collected in Juquiá (650,651,653), Itariri (535), Embu das Artes (524,535) and São Paulo (549, 551). Due to these values we could not assign a clear barcode gap between *B. tenagophila* and *B. occidentalis* and *B. t. guaibensis* (Fig. 2 b, d, f). However, the Bayesian tree inferred from COI data (Fig. 3), as well as the ABGD and both bPTP and GMYC analyses recovered these close related taxa as distinct groups. We suggest that in geographical areas where *B. tenagophila* species complex have the same geographical distribution.

The application of the 3-4% cutoff value for maximum intraspecific divergence may be appropriate for our dataset as 36% of the intraspecific comparisons reached this value (Table 4). The highest values for intraspecific divergence (>3%) do not appear to be a consequence of geographic distance given that the greatest divergence in *B. tenagophila* was between closely proximal localities in São Paulo state (Fig. 3).

*Biomphalaria glabrata* and *B. tenagophila*, are differentiated by the renal ridge, which is present in the former and absent in the latter. Paraense and Deslandes (1959) described a false ridge that runs obliquely to the renal tube and is attached to the pneumostome, in specimens of *B. tenagophila* from Macaé, RJ. The presence of this false ridge in *B. tenagophila* may lead to incorrectly identify this species, particularly in juvenile specimens or specimens that have not been properly fixed. Five specimens of *B. tenagophila* in our study (three from São Lourenço da Serra and two from São Paulo) had a membrane on the renal tubes similar to that described by Paraense and Deslandes. The genetic distance of 9% between *B. glabrata* and *B. tenagophila* observed with both genetic distance and tree-based approaches show that DNA barcoding is an important tool for identifying these closely similar taxa.

The ABGD analysis partitioned *B. glabrata* into five distinct groups, while the GMYC analysis yielded a more cohesive group. Despite the pronounced COI divergence within *B. glabrata*, in all the specimens analyzed here the renal ridge has been considered a robust and consistent taxonomical character, suggesting that morphology is more effective than DNA barcode in this case. However, bBTP analysis and phylogenetic reconstruction supported *B. glabrata* as single and well supported MOTU, a result congruent with the morphological identification.

Another group of morphologically similar and frequently misidentified congeners includes *B. intermedia* and *B. straminea*; the latter a natural intermediate host of *S. mansoni*. Of the seventeen diagnostic characters common to *B. straminea* and *B. intermedia*, the degree of corrugation in the dorsal wall of the vagina has been used to these taxa as a species complex (Paraense 1975) . The vaginal corrugation, which is markedly wavy in *B. straminea* appears as swollen in *B. intermedia*. The large genetic divergence between *B. straminea* and *B. intermedia*, which was 9% greater than the intraspecific values in both species, indicates that these two species can be identified by DNA barcode. Note, however, that our study only included two of the three species in the *B. straminea* complex, as *B. kuhniana* does not occur in São Paulo state (Paraense 1988, Teodoro et al. 2010). In addition, we were unable to collect many specimens of *B. intermedia* owing to its rareness in São Paulo state.

Our findings show *Biomphalaria* species delimitation by phylogenetic approaches and bPTP yielded the same groups identified by traditional taxonomy. The use of DNA barcode to identify species in conjunction with *Biomphalaria* surveys requires the application of both evolutionary and bioinformatics criteria, making it a time-consuming approach that is dependent on specialist knowledge. Morphological identification also requires specialist knowledge. However, as shown in this study DNA barcoding can identify subtle (genetic) differences between intraspecific populations that are not detectable by traditional morphological study.

Furthermore, morphological identification of *Biomphalaria* species depends on subjective interpretation of anatomical variations, as these are measured in terms of relative rather than absolute sizes. We therefore agree with Hebert and Gregory (2005, p. 853), who stated that by reversing the logic of standard taxonomic approaches that “*operate in an a priori fashion—seeking…morphological discontinuities*”, DNA barcoding may, as “*a posteriori approach*”, direct the study of morphological variation in genetically divergent groups of *Biomphalaria*.

## Supplementary materials

Supplementary material 1 Figure S1 Data type: molecular data Explanation note: ML and NJ trees generated with MEGA 6 software from the alignment of the 104 Biomphalaria COI sequences. This dataset is made available under the Open Database License (http://opendatacommons.org/licenses/odbl/1.0/). The Open Database License (ODbL) is a license agreement intended to allow users to freely share, modify, and use this Dataset while maintaining this same freedom for others, provided that the original source and author(s) are credited.Author: Roseli Tuan
