# Supplementary material for: Molecular and morphological identification of Biomphalaria species from the state of São Paulo, Brazil
Source: Zookeys. 2017 Apr 12;(668):11–32. doi: 10.3897/zookeys.668.10562 (PMC5534528; doi:10.3897/zookeys.668.10562)
Supplement: Supplementary material 1 — Figure S1 [file zookeys-668-011-s001.pdf]

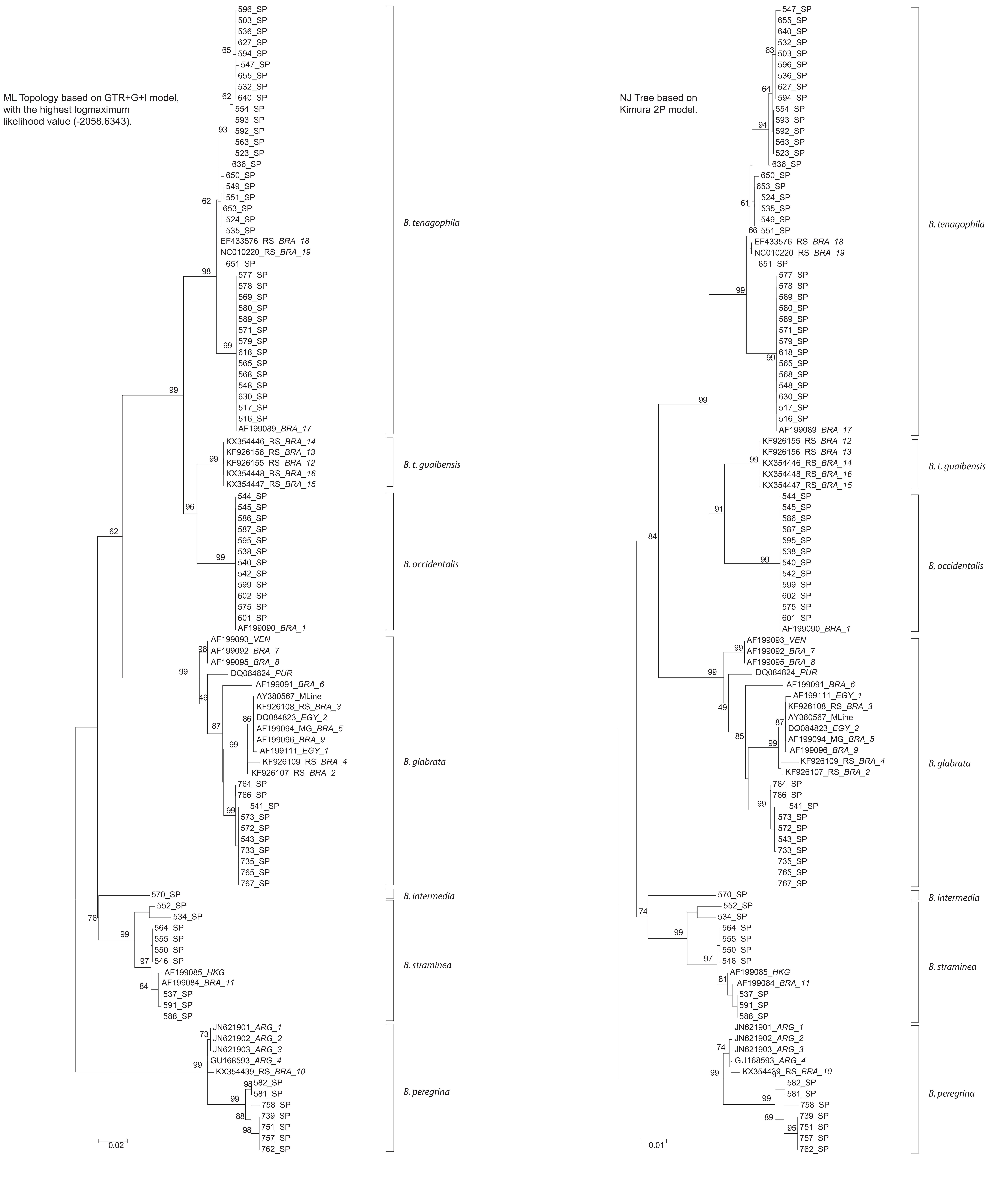

ML Topology based on GTR+G+I model, with the highest logmaximum likelihood value (-2058.6343).
596_SP
503_SP
536_SP
627_SP
594_SP
547_SP
655_SP
532_SP
640_SP
554_SP
593_SP
592_SP
563_SP
523_SP
636_SP
650_SP
549_SP
551_SP
653_SP
524_SP
535_SP
EF433576_RS_BRA_18
NC010220_RS_BRA_19
651_SP
577_SP
578_SP
569_SP
580_SP
589_SP
571_SP
579_SP
618_SP
565_SP
568_SP
548_SP
630_SP
517_SP
516_SP
AF199089_BRA_17
KX354446_RS_BRA_14
KF926156_RS_BRA_13
KF926155_RS_BRA_12
KX354448_RS_BRA_16
KX354447_RS_BRA_15
544_SP
545_SP
586_SP
587_SP
595_SP
538_SP
540_SP
542_SP
599_SP
602_SP
575_SP
601_SP
AF199090_BRA_1
AF199093_VEN
AF199092_BRA_7
AF199095_BRA_8
DQ084824_PUR
AF199091_BRA_6
AY380567_MLine
KF926108_RS_BRA_3
DQ084823_EGY_2
AF199094_MG_BRA_5
AF199096_BRA_9
AF199111_EGY_1
KF926109_RS_BRA_4
KF926107_RS_BRA_2
764_SP
766_SP
541_SP
573_SP
572_SP
543_SP
733_SP
735_SP
765_SP
767_SP
570_SP
552_SP
534_SP
564_SP
555_SP
550_SP
546_SP
AF199085_HKG
AF199084_BRA_11
537_SP
591_SP
588_SP
JN621901_ARG_1
JN621902_ARG_2
JN621903_ARG_3
GU168593_ARG_4
KX354439_RS_BRA_10
582_SP
581_SP
758_SP
739_SP
751_SP
757_SP
762_SP
0.02
B. tenagophila
B. t. guaibensis
B. occidentalis
B. glabrata
B. intermedia
B. straminea
B. peregrina
NJ Tree based on Kimura 2P model.
547_SP
655_SP
640_SP
532_SP
503_SP
596_SP
536_SP
627_SP
594_SP
554_SP
593_SP
592_SP
563_SP
523_SP
636_SP
650_SP
653_SP
524_SP
535_SP
549_SP
551_SP
EF433576_RS_BRA_18
NC010220_RS_BRA_19
651_SP
577_SP
578_SP
569_SP
580_SP
589_SP
571_SP
579_SP
618_SP
565_SP
568_SP
548_SP
630_SP
517_SP
516_SP
AF199089_BRA_17
KF926155_RS_BRA_12
KF926156_RS_BRA_13
KX354446_RS_BRA_14
KX354448_RS_BRA_16
KX354447_RS_BRA_15
544_SP
545_SP
586_SP
587_SP
595_SP
538_SP
540_SP
542_SP
599_SP
602_SP
575_SP
601_SP
AF199090_BRA_1
AF199093_VEN
AF199092_BRA_7
AF199095_BRA_8
DQ084824_PUR
AF199091_BRA_6
AF199111_EGY_1
KF926108_RS_BRA_3
AY380567_MLine
DQ084823_EGY_2
AF199094_MG_BRA_5
AF199096_BRA_9
KF926109_RS_BRA_4
KF926107_RS_BRA_2
764_SP
766_SP
541_SP
573_SP
572_SP
543_SP
733_SP
735_SP
765_SP
767_SP
570_SP
552_SP
534_SP
564_SP
555_SP
550_SP
546_SP
AF199085_HKG
AF199084_BRA_11
537_SP
591_SP
588_SP
JN621901_ARG_1
JN621902_ARG_2
JN621903_ARG_3
GU168593_ARG_4
KX354439_RS_BRA_10
582_SP
581_SP
758_SP
739_SP
751_SP
757_SP
762_SP
0.01
B. tenagophila
B. t. guaibensis
B. occidentalis
B. glabrata
B. intermedia
B. straminea
B. peregrina
